# The coalition of the willing: A deliberate leadership strategy for reforming postgraduate emergency medicine assessment in South Africa

**DOI:** 10.4102/jcmsa.v4i1.419

**Published:** 2026-07-20

**Authors:** Sa’ad Lahri

**Affiliations:** 1 Division of Emergency Medicine, Faculty of Medicine and Health Sciences, Stellenbosch University, Cape Town, South Africa

**Keywords:** leadership, competency-based medical education, assessment reform, emergency medicine, change management

## Abstract

This commentary reflects on two consecutive terms as President of the College of Emergency Medicine of South Africa and examines a deliberate leadership strategy used to reform postgraduate assessment within a voluntary professional body. At the outset, the postgraduate emergency medicine curriculum required reform, and existing assessment approaches had developed over time in ways that were not always fully aligned with evolving training goals or contemporary validity expectations. As competency-based medical education and entrustable professional activity frameworks gained traction, the need for closer alignment between curriculum and assessment became more pressing. Rather than framing the challenge as resistance, this account conceptualises it as a legitimate constraint, encompassing limited faculty capacity, uneven institutional readiness and competing clinical demands. Drawing on adaptive leadership and change management theory, the article argues that sustainable reform may depend less on achieving broad consensus and more on mobilising those already prepared to act. A ‘coalition of the willing’, composed largely of early-career specialists with expertise in competency-based education and digital systems, enabled both curriculum reform and alignment with assessment, alongside key changes including digital examination processes and a standardised objective structured clinical examination across training institutions. This approach was balanced by deliberate strategies to maintain institutional cohesion through ongoing engagement with governance structures. The commentary argues that mobilising readiness is a principled leadership response and that meaningful reform requires acting within constraint while preserving institutional cohesion. In this balance lies the work of leadership in complex professional systems.

## Introduction

The case for reforming health professions education ultimately depends on patient outcomes. The 2010 Lancet Commission argued that health professions education should produce graduates who can strengthen health systems, respond to population needs and promote health equity.^1^ Education systems that do not achieve these aims must change.^1^

In emergency medicine, this matters acutely: a poorly trained emergency physician is a patient safety risk.^2^ However, in South Africa, postgraduate emergency medicine assessment had evolved organically over many years. It reflected a time-based approach that grew harder to defend on validity grounds, sat at odds with the competency frameworks adopted in training, and was delivered unevenly across institutions. Trainees develop as holistic practitioners across multiple domains, and assessment systems need to consistently reflect that breadth.

The move towards entrustable professional activities (EPAs), therefore, offered a practical way to bring curriculum, workplace learning, supervision and assessment into closer alignment.^2,3^ Rather than leaning on time served, clinical exposure and high-stakes summative examinations, EPAs foreground the professional work that trainees must be trusted to perform in practice.^2^ This shift carried particular weight in emergency medicine, where competence includes not only knowledge and procedural skill but also clinical reasoning, prioritisation, communication, leadership, professionalism and safe decision-making under pressure.^3^

This commentary reflects on two consecutive terms as President of the College of Emergency Medicine of South Africa (CEMSA). Like all reflective accounts, this one is shaped by positionality. I write as the person who led the strategy, an insider to CEMSA’s governance with a stake in the reforms described and privileged access to the decisions behind them, but a necessarily partial view of how they were experienced by colleagues, trainees and other institutions. What follows represents the view from a particular leadership role within a complex institutional system, and is offered in that spirit. That caveat stated, the account advances a central claim: in resource-constrained, voluntary professional bodies, reform may depend less on achieving broad consensus and more on deliberately mobilising those who are already ready to act. The argument is not that resistance from established colleagues was unreasonable. Colleagues’ concerns about time, resources and capacity were legitimate and reflected the realities of working in a voluntary professional body. The argument is that sustainable reform cannot wait for universal readiness, and that deliberately mobilising those who are prepared is not a pragmatic shortcut but a principled leadership strategy.

## Context: Convergent pressures on an examining body

College of Emergency Medicine of South Africa, operating under the auspices of the College of Medicine of South Africa (CMSA), holds responsibility for the Fellowship in Emergency Medicine (FCEM) and the Diploma in Primary Emergency Care (DipPEC). These are high-stakes assessments that determine specialist certification and serve as quality assurance for the emergency medicine workforce. The College is also responsible for curriculum oversight, examiner development and standard-setting.

At the outset of the period under reflection, several pressures converged. South Africa had been identified as a country adopting competency-based medical education (CBME) to address healthcare worker shortages.^4^ The Curricular Redesign, Evaluation and Training in Emergency Medicine (CREATE) collaboration, a multi-institutional initiative spanning six South African universities, was actively redesigning the postgraduate emergency medicine (EM) curriculum using EPA frameworks, and the FCEM and DipPEC required corresponding reform to maintain alignment.^4^ Central to this work was the articulation of a defining professional identity for the South African emergency physician: a medical expert, rooted in community through team-based practice, social accountability and context-specific care, demonstrating leadership as a critical thinker, communicator, teacher, professional and manager.^4^ This conception of the graduate provided both the normative anchor for curriculum redesign and the standard against which examination formats could be evaluated.

Examination practice had also developed organically and rested on assumptions about training time that were no longer defensible. Before the coronavirus disease 2019 (COVID-19) pandemic, assessment was largely face-to-face. The pandemic moved examinations online, but the online format had no clinical component. This left a gap: clinical competence at the ‘shows how’ and ‘does’ levels of Miller’s pyramid, where performance is directly observed, was no longer being assessed consistently across institutions.^5^ At the same time, a generation of experienced examiners was approaching the end of active service, while relatively few early-career emergency physicians had been drawn into College governance. These pressures accumulated gradually, each individually manageable yet collectively demanding more than incremental adjustment. Persistent challenges in leadership, governance and human resource capacity have been identified as key constraints to the transformation of health professions education in South Africa.^6^

## The nature of the challenge: Legitimate constraint, not simple resistance

It would be tempting, and inaccurate, to frame the reform challenge as one of overcoming resistance. The concerns raised by programme leaders, supervisors and trainees were not oppositional to reform, but reflected practical anxieties about whether the system was ready to implement it fairly and consistently. These concerns centred on limited staff capacity, uneven faculty development, a lack of protected time for supervision and assessment, variable institutional infrastructure and uncertainty about how workplace-based assessment decisions would be quality-assured across training sites. Ras and colleagues^7^ documented that structural readiness, staff capacity and the social dynamics of supervision are substantive barriers in South African specialist training programmes. Daitz and colleagues^8^ similarly showed that trainees supportive of reform anticipate uneven implementation without protected time, faculty development and context-sensitive quality assurance. Constraint, therefore, rather than resistance, is the dominant feature of this context.

These constraints, however, could not become the ceiling of ambition. The external landscape would not pause while internal readiness caught up. Adaptive leadership theory, developed by Heifetz and colleagues, is a framework for mobilising people to confront complex, ill-defined challenges and to adapt under shifting, uncertain conditions.^9,10^ It distinguishes technical problems, which can be resolved through existing expertise, from adaptive challenges, which call for changes in how we think, what we value and how we behave.^9,10^ Applied here, it helps make sense of the dynamic facing the CEMSA. The challenge was not disagreement about the destination but divergence in judgement about what was feasible now. That is an adaptive problem, not a technical one, and it calls for a different response: mobilising those who are ready.

## The coalition of the willing as a deliberate leadership strategy

Kotter’s^11^ change model identifies the formation of a guiding coalition as foundational. In voluntary professional bodies, however, waiting for coalition membership to represent the full breadth of institutional opinion risks stagnation. The strategic response within CEMSA was to identify early-career specialists who combined CBME fluency, digital capability and commitment to reform, and to centre the work of change around them.

This approach extends Kotter’s model in a specific way: rather than assembling a coalition for representational purposes alone, the strategy deliberately mobilised existing adaptive capacity.^11,12^ The distinction matters. Representation asks who should be at the table. Mobilisation of readiness asks where energy and capability already reside, and organises around those concentrations. In this instance, the judgement about where energy and capability resided was made by the College President, together with the leadership of the CREATE group. It was based on direct knowledge of colleagues at local universities who had already shown interest in the reform process, attended discussions, contributed ideas and demonstrated a willingness to participate in curriculum, assessment and workplace-based assessment reform. These individuals were not selected only because they formally represented particular institutions, but because they had already shown readiness to contribute and to help move the reform forward from within their own training contexts. In a voluntary body with limited discretionary capacity, these are not the same question, and conflating them can immobilise reform. The strategic selection of who moves first is not, in this framing, an interim compromise. It is a defining leadership act.

The coalition delivered the core architecture of the reform. Its work included the development of a new curriculum built around a structured set of EPAs, a revised examination blueprint that mapped assessment systematically onto that curriculum and digital tools to capture EPAs in practice. Alongside these curriculum and workplace-based assessment reforms, the group introduced a standardised objective structured clinical examination (OSCE) across all training institutions, creating a shared format for assessing clinical competence, where previous approaches had varied across sites. This work was not developed in isolation. Coalition members returned to their universities to seek feedback from registrars and faculty, and the design was adapted in response.

The significance of the work lay not only in its technical execution but in what it demonstrated: that reform of this scale was feasible. By making change visible and concrete, the coalition created the conditions for broader institutional engagement. Early-career specialists thus proved to be a present rather than a future resource for reform, particularly in the CBME and digital assessment domains.^5^ Within CEMSA, their involvement reframed what was institutionally possible.

Institutional endorsement remained essential. College of Medicine of South Africa leadership’s support ensured that coalition-driven work remained aligned with formal governance structures and did not operate as a parallel authority.

## Holding space for late adopters

A coalition strategy carries a risk that must be acknowledged: the emergence of a two-speed organisation, in which a technically agile group moves forward while others are left behind or perceive themselves to be. In a voluntary professional body, a coalition driving reform may also be seen as operating ahead of elected governance structures, creating a legitimacy problem that can undermine the very reforms it produces.

Managing these tensions required deliberate effort. Regular feedback sessions with the CEMSA council ensured ongoing engagement with both the direction and detail of reform.

Overlapping membership between coalition working groups and CEMSA council structures created continuity and reduced scope for divergence. The universities were also essential partners in this work. As the sites where training and assessment actually take place, their faculty and training committees were engaged throughout, ensuring that reforms were tested against institutional realities and owned beyond the coalition itself. Work was consistently framed as contributing to shared institutional purpose, with emphasis on distributed workload rather than concentrated influence. These measures were not merely procedural. They reflected a substantive leadership principle: that the pace of progress must be calibrated not only by what is technically possible but by what the institution can sustain as coherent.^13^

This reflects a core tension in leading change in complex professional bodies: the need to move at the pace of possibility without undermining the institutional cohesion on which durable reform depends.

## Insights for leaders of professional bodies

Several transferable insights emerge:

Firstly, legitimate constraint and readiness for reform are not synonymous. Conflating them risks paralysing necessary change by treating reasonable caution as an obstacle rather than a condition to be managed.

Secondly, early-career specialists represent a present resource for reform in CBME and digital assessment domains, and their inclusion in governance is a strategic rather than merely developmental decision.^6^

Thirdly, coalition-building in voluntary bodies depends on credibility, consistency and visible institutional endorsement. A coalition lacking formal legitimacy produces outputs that can be undone.

Fourthly, assessment reform must align with curricular reform. Misalignment between what is taught and what is assessed produces incoherence that trainees and examiners recognise, and that ultimately undermines both enterprises.

Fifthly, digital capability is now integral to examination quality, and its uneven adoption within examining bodies is itself a validity concern.

## Conclusion

Leading reform within CEMSA required navigating the gap between necessity and feasibility without allowing that gap to become permanent. The coalition of the willing offered a way forward that was neither impatient with legitimate constraint nor paralysed by it. The purpose, ultimately, was not organisational efficiency. It was better-trained emergency physicians and safer patient care: the same imperative the 2010 Lancet Commission placed at the centre of every argument for reforming how health professionals are educated.^1^

This reflects a broader paradox in leading change within complex professional systems: progress often requires action before consensus is achieved, while simultaneously preserving the conditions that make future consensus possible. Managing that paradox, rather than dissolving it, may be the central task of institutional leadership in health professions education.

The work, however, is not finished. Reform is not an event but a discipline. Assessment systems require continuous scrutiny, regular review against evolving curricular ambitions, and honest evaluation of whether what is being measured reflects what matters most for patient care. The coalition that enabled change must become the culture that sustains it. For those leading professional bodies in emergency medicine and beyond, the challenge is not only to build what was missing, but to remain committed to questioning what has been built. In that ongoing commitment, not in any single achievement, lies the true measure of institutional leadership.

## References

[1] Frenk J, Chen L, Bhutta ZA, et al. Health professionals for a new century: Transforming education to strengthen health systems in an interdependent world. Lancet. 2010;376(9756):1923–1958. 10.1016/S0140-6736(10)61854-5 21112623

[2] Teng D, Venkataraman A, Singer A, et al. Entrustable professional activities for emergency medicine specialists. Int J Emerg Med. 2025;18(1):248. 10.1186/s12245-025-01075-z 41291421 PMC12645698

[3] Seetharaman R. Pros and cons: Global adoption of competency-based medical education. Acad Med. 2023;98(12):1346. 10.1097/ACM.0000000000005453 37683269

[4] College of Medicine of South Africa (CMSA). FCEM living curriculum and WBA update [homepage on the Internet]. Johannesburg: CMSA; 2024 [cited 2026 Mar 30]. Available from: https://cmsa.co.za/wp-content/uploads/2024/02/FCEM_Living_Curriculum_and_WBA_update_15_2_2024.pdf

[5] Miller GE. The assessment of clinical skills/competence/performance. Acad Med. 1990;65(9 suppl):S63–S67. 10.1097/00001888-199009000-00045 2400509

[6] Von Pressentin KB, De Sá A, Pampallis P, Ras T. Cultivating leaders for primary health care: A revised approach for transformative development. Afr J Prim Health Care Fam Med. 2024;16(1):e1–e4. 10.4102/PHCFM.v16i1.4410 PMC11079346 38708732

[7] Ras T, Stander Jenkins L, Lazarus C, et al. ‘We just don’t have the resources’: Supervisor perspectives on introducing workplace-based assessments into medical specialist training in South Africa. BMC Med Educ. 2023;23(1):832. 10.1186/s12909-023-04840-x 37932732 PMC10629100

[8] Daitz E, Jenkins LS, Van Rensburg JJ, et al. The introduction of workplace-based assessment into postgraduate medical training in South Africa: Trainee perspectives. BMC Med Educ. 2026;26(1):515. 10.1186/s12909-026-08792-w 41723383 PMC13032551

[9] Heifetz RA, Linsky M, Grashow A. The practice of adaptive leadership: Tools and tactics for changing your organization and the world. Boston, MA: Harvard Business Press; 2009.

[10] McKimm J, Ramani S, Forrest K, et al. Adaptive leadership during challenging times: Effective strategies for health professions educators: AMEE Guide No. 148. Med Teach. 2023;45(2):128–138. 10.1080/0142159X.2022.2057288 35543323

[11] Kotter JP. Leading change. Boston, MA: Harvard Business School Press; 1996.

[12] Graves L, Dalgarno N, Hoorn RV, et al. Creating change: Kotter’s change management model in action. Can Med Educ J. 2023;14(3):136–139. 10.36834/cmej.76680 PMC10351637 37465754

[13] Karimi E, Sohrabi Z, Aalaa M. Change management in medical contexts, especially in medical education: A systematized review. J Adv Med Educ Prof. 2022;10(4):219–227. 10.30476/JAMP.2022.96519.1704 36310665 PMC9589067

